# Editorial Discussion of Portal Hypertension, Variceal Bleeding and High Output Cardiac Failure Secondary to an Intrahepatic Arterioportal Fistula

**DOI:** 10.1155/1993/49139

**Published:** 1993

**Authors:** Layton F. Rikkers, Merle M. Musselman

**Affiliations:** Department of Surgery University of Nebraska Medical Center 600 South 42nd Street Omaha Nebraska 68198-3280 USA


*HPB Surgery*, 1993, Vol. 7, pp. 51–52
Reprints available directly from the publisher
Photocopying permitted by license only

© 1993 Harwood Academic Publishers GmbH
Printed in the United States of America

### EDITORIAL DISCUSSION OF

### PORTAL HYPERTENSION, VARICEAL BLEEDING AND HIGH OUTPUT CARDIAC FAILURE SECONDARY TO AN INTRAHEPATIC ARTERIOPORTAL FISTULA

LAYTON F. RIKKERS and MERLE M. MUSSELMAN
*Department of Surgery, University of Nebraska Medical Center, 600 South 42nd Street, Omaha, Nebraska 68198-3280, USA*

### DISCUSSION

Oishi and co-workers present an interesting case of an intrahepatic arterioportal fistula (APF) secondary to blunt abdominal trauma. This is a particularly educational case because the patient developed most of the potential complications of an APF, notably portal hypertension with both bleeding varices and ascites, congestive heart failure secondary to a hyperdynamic state, and a hepatic artery aneurysm.

Although this fistula was secondary to abdominal trauma, most APFs are secondary to percutaneous liver biopsy performed in patients with liver disease. Most are asymptomatic and first detected when evaluating a variceal bleeder with ultrasound or angiography. Since many of these individuals have coexisting cirrhosis, which was the reason for performance of the liver biopsy, it is often difficult to determine whether the APF significantly contributes to the portal hypertension and whether subsequent variceal hemorrhage could be prevented by treatment of the APF alone. In our limited experience, we have seen two patients with cirrhosis who continued to bleed from varices despite successful embolization of an APF. Both of these individuals underwent chronic sclerotherapy which prevented rebleeding in one, but the other patient failed sclerotherapy and eventually required portal decompression. A selective shunt (distal splenorenal shunt) was chosen for that patient because the prior embolization had interrupted a fraction of the hepatic arterial blood supply. We thought it essential to preserve portal flow to avoid infarction of hepatic parenchyma. Because of this potential problem, it is probably preferable to ignore incidentally discovered APFs in variceal bleeders with cirrhosis unless the APF is very large.

Although hepatic arterial embolization by interventional radiologic techniques is probably the most common approach in treating symptomatic APFs, the patient described by Oishi and co-authors required open surgery because of the size of the

Address correspondence to: Layton F. Rikkers, M.D., Department of Surgery, University of Nebraska Medical Center, 600 S. 42nd Street, Omaha, NE 68198-3280, USA. Tel. (402) 559-6719

APF and the presence of a large hepatic artery aneurysm which also required attention. The authors' approach, which consisted of right hepatic lobectomy, resection of the hepatic artery aneurysm and arterial revascularization of the left hepatic lobe, was both innovative and successful in relieving portal hypertension and the systemic hyperdynamic state.

